# Phase one of a hybrid effectiveness-implementation study to assess the feasibility, acceptability and effectiveness of implementing seasonal malaria chemoprevention in Nampula Province, Mozambique

**DOI:** 10.1186/s12936-024-05229-x

**Published:** 2025-02-21

**Authors:** Kevin Baker, Ivan Alejandro Pulido Tarquino, Pedro Aide, Craig Bonnington, Christian Rassi, Sol Richardson, Chuks Nnaji, Arantxa Roca-Feltrer, Maria Rodrigues, Mercia Sitoe, Sonia Enosse, Caitlin McGugan, Francisco Saute, Gloria Matambisso, Baltazar Candrinho

**Affiliations:** 1 Malaria Consortium, London, UK; 2 Department of Global Public Health, Karolinska Institute, Stockholm, Sweden; 3 Malaria Consortium, Maputo, Mozambique; 4 National Institute of Health (Instituto Nacional de Saúde), Maputo, Mozambique; 5 Centro de Investigação em Saúde de Manhiça, Manhiça, Mozambique; 6 Vanke School of Public Health, Tsinghua University, Beijing, China; 7 PATH, Maputo, Mozambique; 8 GiveWell, San Francisco, USA; 9 National Malaria Control Programme, Ministry of Health, Maputo, Mozambique

**Keywords:** Seasonal malaria chemoprevention Mozambique children

## Abstract

**Background:**

Seasonal malaria chemoprevention (SMC) is a highly effective intervention for malaria prevention in high burden areas with seasonal transmission, historically implemented in the Sahel. Mozambique contributes to 4% of global malaria cases. Malaria Consortium, in partnership with the National Malaria Control Programme, conducted a two-year phased SMC study in Nampula province using sulfadoxine-pyrimethamine (SP) plus amodiaquine (AQ), or SPAQ, in children under five. Phase one results presented here highlight acceptability, feasibility, and protective effect of SMC.

**Methods:**

A pragmatic type II hybrid effectiveness-implementation study design was adopted, using mixed methods. The study was conducted in three districts, utilizing: (1) non-randomized controlled trial reporting on malaria incidence; (2) drug resistance molecular marker study reporting on resistance marker changes over time; (3) coverage and quality assessment on the SMC distribution; and (4) a qualitative acceptability and feasibility assessment with stakeholders.

**Results:**

Children who received SMC had 86% (hazard ratio 0.14, 95% CI 0.09–0.24) lower hazards of developing clinical malaria during the peak transmission season compared with children in the comparison district. Prevalence of SP molecular markers associated with resistance was high at baseline (K540E 66.1%). SMC achieved high coverage of eligible children over four cycles (87.7%, 95% CI 83.9–90.8%). Qualitative results indicate SMC was positively accepted by the targeted community.

**Conclusions:**

Results suggest that SMC was effective at preventing clinical malaria, did not significantly impact resistance profile, and was feasible and acceptable in the context. Phase two will assess SMC impact in reducing malaria incidence and if chemoprevention efficacy of SPAQ is impacted by drug resistance and drug concentrations.

## Background

Seasonal malaria chemoprevention (SMC) is a highly effective community-based intervention to prevent malaria infections caused by *Plasmodium falciparum* in areas where the burden of malaria is high, and transmission is seasonal [1]. It involves the intermittent administration of anti-malarial medicines to at-risk populations during the peak malaria season, which typically coincides with the rainy season. The objective is to maintain therapeutic anti-malarial drug concentrations in the blood throughout the period of greatest malarial risk. The World Health Organization (WHO) recommended SMC as a malaria prevention strategy for children 3–59 months since 2012 [2]. The recommendation called for the use of a combination of sulfadoxine-pyrimethamine (SP) and amodiaquine (AQ). Annual SMC rounds comprising 4 monthly SMC cycles were recommended in areas where more than 60% of clinical malaria cases annually occur during a period of 4 months. SMC was not recommended in areas where the therapeutic efficacy of SPAQ is below 90 percent due to resistance among circulating parasites. For this reason, the Sahel region of west and central Africa has been prioritized for the scale-up of SMC, as resistance to SP is widespread across east and southern Africa [3]. More recently, WHO published consolidated guidelines for malaria, which no longer prescribe the number of SMC cycles, age-range or therapeutic efficacy threshold for the deployment of SMC [1].

In clinical trials, SMC was found to prevent 75% of uncomplicated and severe malaria cases in children under five [4]. In the Sahel, it has been demonstrated that SMC implementation at scale achieving high coverage through national health systems and was safe and feasible [3]. Case–control studies in seven countries showed an average protective effectiveness of SMC under programmatic conditions of 88% against clinical malaria [5]. The weighted average economic cost of administering 4 monthly SMC cycles was estimated at $3.63 per child [6]. After 10 years of SMC implementation in the Sahel, there have been increasing calls to explore the use of this successful intervention in new geographies, including areas in east and southern Africa where malaria transmission is highly seasonal [7].

**Fig. 1:**
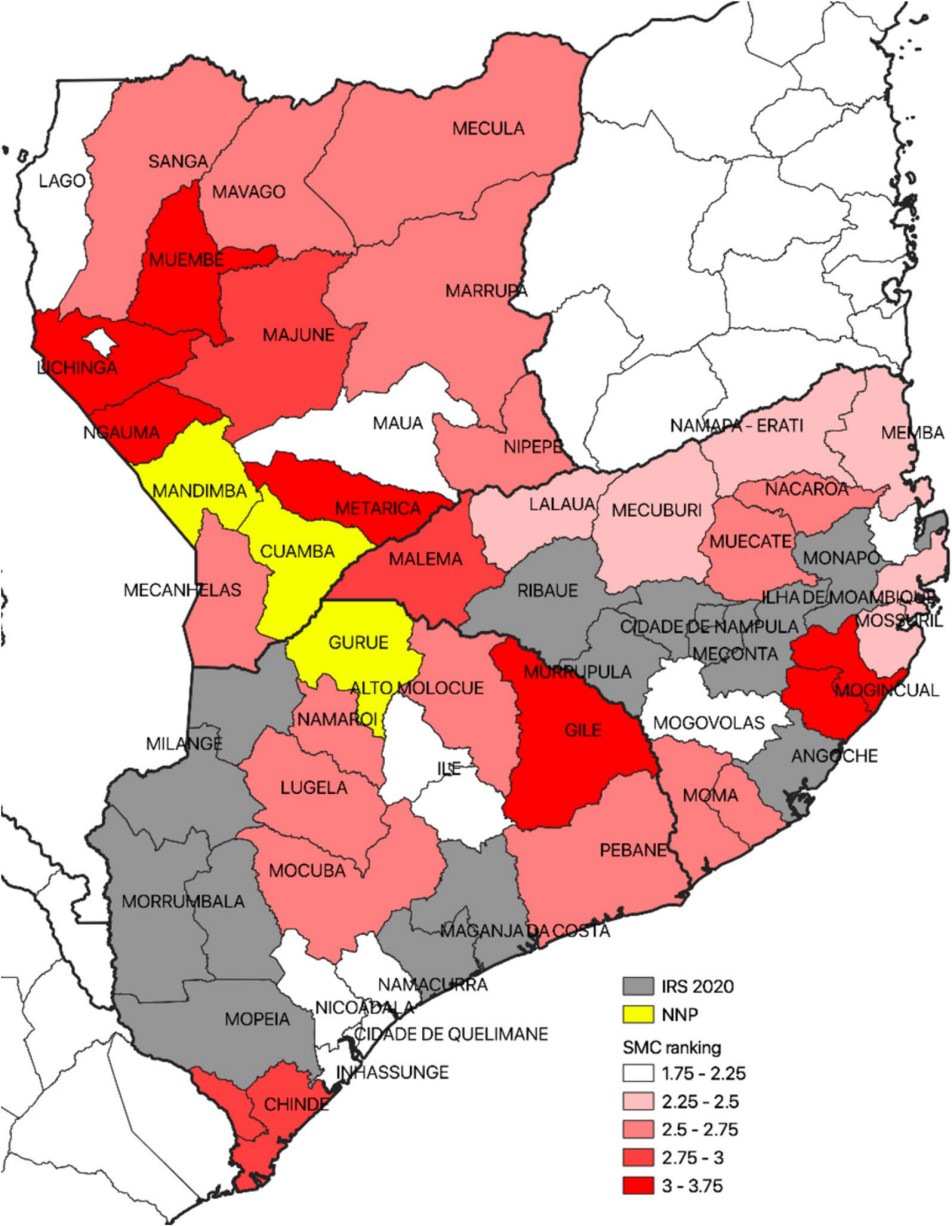
Mozambique SMC modelling exercise

Mozambique accounts for 4% of global malaria deaths [8] and the disease is highly endemic in many areas of the country, with the highest prevalence in the north and along the coast [9, 10]. A mid-term review of the country’s Malaria Strategic Plan 2017–2022 recommended SMC as a strategy to decrease malaria cases in the highest-burden locations [11]. To assess whether SMC could be an effective malaria prevention strategy in an area where resistance to SP is assumed high, Malaria Consortium, in partnership with the Mozambican National Malaria Control Programme (NMCP), initiated a phased SMC implementation project in Nampula province, where under-five mortality is high and malaria transmission is seasonal. This province was selected based on a ranking exercise conducted nationally, which ranked the suitability of different districts based on rainfall, case load, etc. (Fig. 1).

The project was designed as a two-year hybrid effectiveness-implementation study, with first phase focusing on acceptability, feasibility, and the protective effect of SPAQ when used in SMC [12], followed in phase 2 by more rigorous assessments of the effectiveness of the intervention and chemoprevention efficacy of SPAQ [13], which included a clustered-randomized controlled trial (cRCT) and a chemoprevention efficacy study [14]. This two-stage design was recommended by the scientific advisory committee convened to support this work and supporting the evidence requirement from the national malaria control programme. This paper describes results from the first phase of the study.

### Study aims and objectives

The study had two primary aims: to determine the protective effect of SPAQ when used for SMC in the context of northern Mozambique, and to assess the feasibility and acceptability of implementing SMC in terms of coverage, quality, and stakeholder perceptions. Objectives included: (1) to determine whether receipt of SPAQ is associated with a reduction in odds of clinical malaria, (2) to estimate baseline prevalence of SP and AQ resistance markers and measure any increase after one annual round of SMC, (3) to evaluate SMC implementation in terms of quality and coverage, and (4) to explore and acceptability of SMC and implementation feasibility among stakeholders.

## Methods

### Study site

**Fig. 2:**
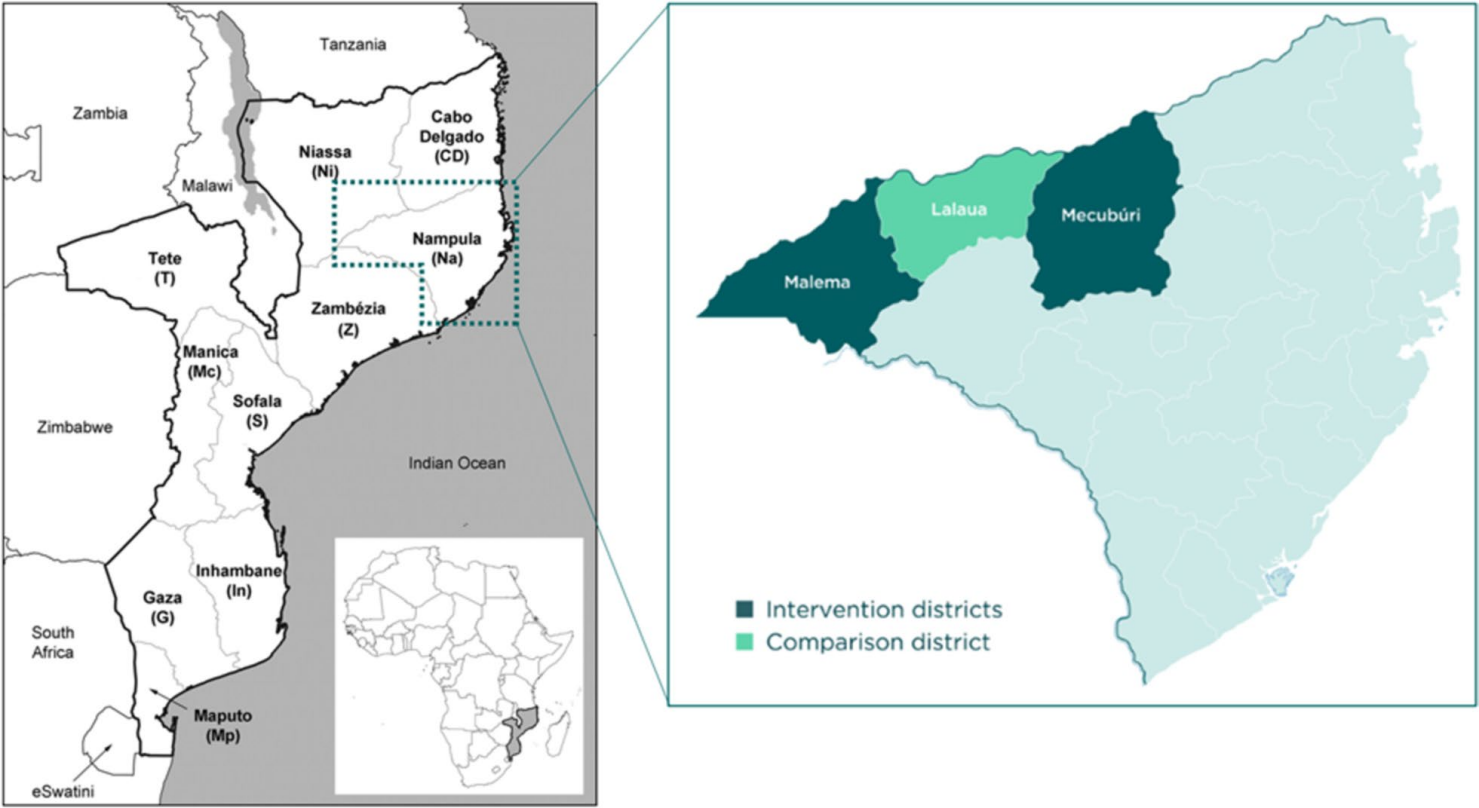
SMC intervention and comparison districts of Nampula Province, Mozambique

The study was conducted in Malema, Mecubúri and Lalaua districts in Nampula province, northern Mozambique (Fig. 2). To identify suitable districts for SMC, a suitability ranking was conducted for all districts. Criteria included in the ranking score are described elsewhere [12].

### Study design

The study was conducted between November 2020 and February 2021. It involved delivering 4 monthly SMC cycles using SP and AQ to a target population of around 72,000 children in two districts of Nampula province: Malema and Mecubúri. SMC delivery followed the standard door-to-door delivery model of 3 days of SPAQ, commonly used in Sahelian countries, with trained volunteers acting as community distributors, supervised by health facility workers. A third district, Lalaua, where SMC was not implemented, served as a comparison area (Fig. 1). A three districts had similar geographical characteristics and malaria interventions, with perennial transmission with distinct seasonal peaks, meaning at least 60% of the total malaria burden occurs within a 5-month period. Lalaua was purposively selected as comparison district based on logistics and sample size requirements.

A non-randomized controlled trial (nRCT) was conducted to calculate the effectiveness of SMC with SPAQ at preventing clinical malaria. To understand the baseline context and estimate whether SMC with SPAQ affected the prevalence of molecular markers associated with drug resistance to a cross-sectional study involving the collection of samples at baseline (before the SMC round) and endline (after the SMC round) was conducted in both the intervention and comparison areas. To estimate coverage and assess quality of delivery of the intervention, a cross-sectional study was conducted, which involved an end-of-round (EoR) household survey. To assess acceptability and feasibility of SMC among key stakeholders, was performed a qualitative study involving focus group discussions and interviews with key stakeholders [12].

### Study population

SMC-eligible children included afebrile children of either gender, aged 3–59 months, residing in Malema and Mecubúri districts. For the EoR survey, children aged 3–119 months were eligible for the study to permit estimation of the extent to which SMC medicines were administered to children outside of the eligible age range. Health workers involved in SMC implementation, caregivers of children under 10 years of age, community leaders and key stakeholders such as health officials at different levels of the health system and those involved in SMC implementation were included in the qualitative study population.

### Primary and secondary outcomes

The primary outcome for the non-randomized controlled trial was malaria incidence reported through optimized passive surveillance. For the resistance markers study, the primary outcome was the prevalence of relevant SPAQ molecular markers associated with anti-malarial drug resistance.

### Non-randomized controlled trial

#### Sample size

A total sample size of 800 children (400 in each arm) was selected to provide sufficient statistical power to have an 80% power of detecting a 40% difference in the odds of clinical malaria cases between children in the intervention and comparison districts, with statistical significance at the 5% level under the assumptions of 0.2 clinical episodes per child per high-transmission season in absence of SMC delivery and 20% loss to follow-up.

In Malema and Lalaua districts, the same number of settlements were selected, using a simple random procedure. Within settlements, compounds were randomly sampled by researchers using household lists from selected communities, with one eligible child aged 3–59 months recruited at random in each household for study enrolment. Children were recruited in 63 clusters in total, with a target of 15 children in each. Upon recruitment, a short baseline questionnaire was administered to collect individual- and household-level data and confirm their eligibility. The analysis compared the hazard of development of rapid diagnostic test (RDT)-confirmed cases of malaria among eligible children during the follow-up after each four SMC rounds comparing an intervention district (Malema) with SMC delivery to a comparison district (Lalaua) without SMC delivery. In the intervention district, a researcher followed each community distributors as they administered SMC in each cycle and queried children’s caregivers on incidence of malaria in the selected child, as well as visits to health facilities. If caregivers reported a case of malaria, researchers referred to records at local clinics for RDT confirmation of cases. If children enrolled in the study were experiencing a fever (> 37.5 °C) at the time of the community distributors’ visit, they were referred to their local health facility and tested using an RDT. In the event of a positive test outcome, the dates of malaria case confirmation were recorded on a clinic logbook entry. In the comparison district, the same outcome reporting system was followed. The febrile children at the first visit were referred to the community health worker if available or to the nearest health facility (HF). Community health workers were the link between community and HF. A community health worker (CHW) can serve one or more communities. They can perform a malaria RDT and provide treatment. Data of children presented were recorded in the CHW’s logbook, which was integrated in the HF logbooks on weekly basis. These logbooks have the same indicators which facilitated encoding of information. Research team in the field had a constant communication with the CHWs in order to inform them about specific situation and the need to follow up of febrile children.

### Data analysis

Baseline study participant characteristics were described and checked for comparability between arms by using Chi square. The proportion of children in each arm who experienced at least one RDT-confirmed malaria case during the follow-up period was summarized and statistically significant differences between intervention and comparison districts determined by using a Pearson Chi square test. Exact odds ratios (ORs) were calculated for the effect of SMC delivery. To estimate effect of SMC treatment using hazard ratios (HRs) two Cox proportional hazards models were fitted: a standard Cox proportional hazards model for time to failure (defined as a participating child’s first RDT-confirmed malaria case) with right censoring in the event of loss to follow-up (Model 1), and a random-effects Cox proportional hazards models (with random intercepts for individual children) (Model 2) for recurrent malaria cases and multiple periods of follow-up. If recurrent events were recorded, children who experienced an RDT-confirmed malaria case were considered to have ‘recovered’ the day following case confirmation and were considered to have started a new follow-up period. If a child could not be found by researchers during any of the follow-up visits, they were considered lost to follow-up for the preceding period since the baseline survey or previous SMC cycle date but were considered to have started a new follow-up period if they were subsequently re-located and returned to follow-up. To adjust for local differences in hazard of malaria incidence, Model 2 was extended by fitting random intercepts for settlements to account for area-level clustering of risk of experiencing an RDT-confirmed malaria case (Model 3). The child’s sex, age (as a categorical variable), net use at baseline, use of other preventive measures against malaria at baseline, wealth index (as a continuous variable), and receipt of Day 2 and Day 3 AQ in the previous month’s SMC cycle were covariates selected for inclusion in the models. Variables were selected for inclusion using forwards stepwise selection based on Collett’s method [15] and were retained if they were found to significantly improve model fit as determined using the likelihood ratio test. Data were analysed using Stata 17.0. The study was reported in accordance with the Strengthening the Reporting of Observational Studies in Epidemiology (STROBE) guidelines.

### Drug resistance molecular markers study

Trends in molecular markers of SP and AQ resistance were monitored in the three districts. A health facility-level cross-sectional survey was conducted in October 2020, before the start of SMC implementation (baseline) and 28 days after the end of SMC distribution (endline) in March 2021. Blood samples were collected on filter paper (dried blood spots) from children aged less than 5 years with a positive RDT, that is evidence of *Plasmodium falciparum* infection, attending selected health facilities in the intervention and comparison areas. Four health facilities were selected in the intervention districts (two in Malema and two in Mecubúri) and in four health facilities in the comparison district (Lalaua). All health facilities were purposely selected based on their contribution to the total number of cases.

To identify molecular markers of resistance to anti-malarial, DNA extraction and nested-PCR for genotyping were carried out in the molecular biology laboratory of the Manhica Health Research Centre (CISM). Then, the amplicons were sent for sequencing using the Sanger method, by Macrogen (https://dna.macrogen-europe.com/eng/support/ces/guide/troubleshooting.jsp).

The key markers included: dihydrofolate reductase (*dhfr*): codons 108, 51, 59 and 164; dihydropteorate synthetase (*dhps*): codons 431, 437, 540, 581 and 613; *P. falciparum* chloroquine resistance transporter gene (*pfcrt*): codons 72–76; and *P. falciparum* multidrug resistance gene 1 (*pfmdr1*): codons 86, 184 and 1246. Molecular procedures are detailed elsewhere [12]. *Plasmodium falciparum* DNA from MR4 (the malaria research and reference reagent source centre) were included as negative and positive controls for each gene: MRA-151G genomic DNA from *P. falciparum* 3D7A (*dhfr* Wild-type; *dhps* A437G); MRA-731G genomic DNA from *P. falciparum* FCR-3/Gambia [Subline F-86] (*dhfr* S108T; *dhps* wildtype); MRA-150G genomic DNA from *P. falciparum* Dd2 (*pfcrt* M74I N75E K76T A220S Q271E N326S I356T R371I; *pfmdr1* N86Y I1034S) and MRA-102G genomic DNA from *P. falciparum* 3D7 (*pfcrt* wild-type; *pfmdr1* wild-type).

#### *End-of-round survey*

An end-of-round cluster cross-sectional survey was conducted following delivery of cycle 4 to assess key indicators including the proportion of eligible children who received Day 1 SPAQ in cycle 4, receipt of Day 1 SPAQ with adherence of distributors to directly observed therapy (DOT) in cycle 4, caregiver adherence to administration of AQ on Day 2 and Day 3 in cycle 4, the proportion of eligible children who received Day 1 SPAQ in all four cycles, and the proportion of ineligible older children aged 60–119 months who received Day 1 SPAQ in cycle four. The sample size calculations are described elsewhere [12]. Across both districts, settlements were selected with probability proportional to their population size to give a self-weighting sample that was representative of the overall population of the two districts. The survey sampled 90 settlements with 10 households in each, randomly selecting residential structures (comprising either single-family residences or multi-family compounds) from lists of residential structures with at least one child aged 3–119 months until a sample size of 900 was reached. Household surveys were administered using SurveyCTO version 2.71. In each structure, a roster of all children aged 3–119 months was compiled; one child was selected at random from the roster by SurveyCTO and all questions on SMC indicators related to that child, their caregiver and household. In addition to key SMC indicators, data on a range of other variables relating to children, caregivers and households was collected. The analytic sample for analysis of key indicators excluded children who were ineligible for SMC administration in cycle 4 for any reason other than age (known allergy to SP or AQ, or fever at the time of household visits by distributors). Survey methods are described elsewhere [16].

#### SMC acceptability and feasibility

20 focus group discussions (FGDs) in Malema and Mecubúri districts, and 20 key informant interviews (KIIs) at district and national level were conducted to assess acceptability and feasibility of SMC. FGDs were held with caregivers of children who received SMC, community distributors and health workers (supervisors of community distributors). Community leaders and stakeholders involved in SMC implementation at national, district and provincial level with malaria knowledge and experience were selected as participants in KIIs. Participants were selected through purposive sampling to ensure a wide range of views on SMC. Participants for FGD were recruited based on their presence at home during the enrolment in the study and distribution of SPAQ during the four cycles. Key informants were recruited based on their role during the SMC campaign at any level from community to central level. All participants were provided with information on the study and granted time to clarify any emerging questions. Data were collected using semi-structured interviews. Data collection tools were pre-tested prior to implementation. KIIs and FGDs were audio recorded, transcribed, and translated from Emakhuwa (local language) to Portuguese by two researchers fluent in both languages. Transcripts were uploaded into MAXQDA qualitative software for analysis. Through thematic content analysis [17], codes were identified and organized manually into categories and major themes. Two co-investigators compared their findings and discussed areas of agreement as well as areas of divergence during interim and final analysis. Data analysis was conducted in Portuguese and selected quotes representing the identified codes and themes were translated into English.

### Ethical considerations

Ethical approval for this study was received from the Comité Nacional de Bioética para a Saúde (CNBS) of the Ministry of Health of Mozambique on 15 September 2020 (Ref: 508/CNBS/20). Only participants who met the inclusion criteria and whose caregivers provided written informed consent were included in the study.

### Patient involvement

Children who had previously had SMC and their families were not involved in setting the research question, outcome measures or the intervention design, but they were involved in the implementation of the intervention. Communities where SMC was distributed were also central to dissemination of the study results, which helped to motivate community involvement during and beyond the study.

## Results

### Non-randomized controlled trial

**Fig. 3:**
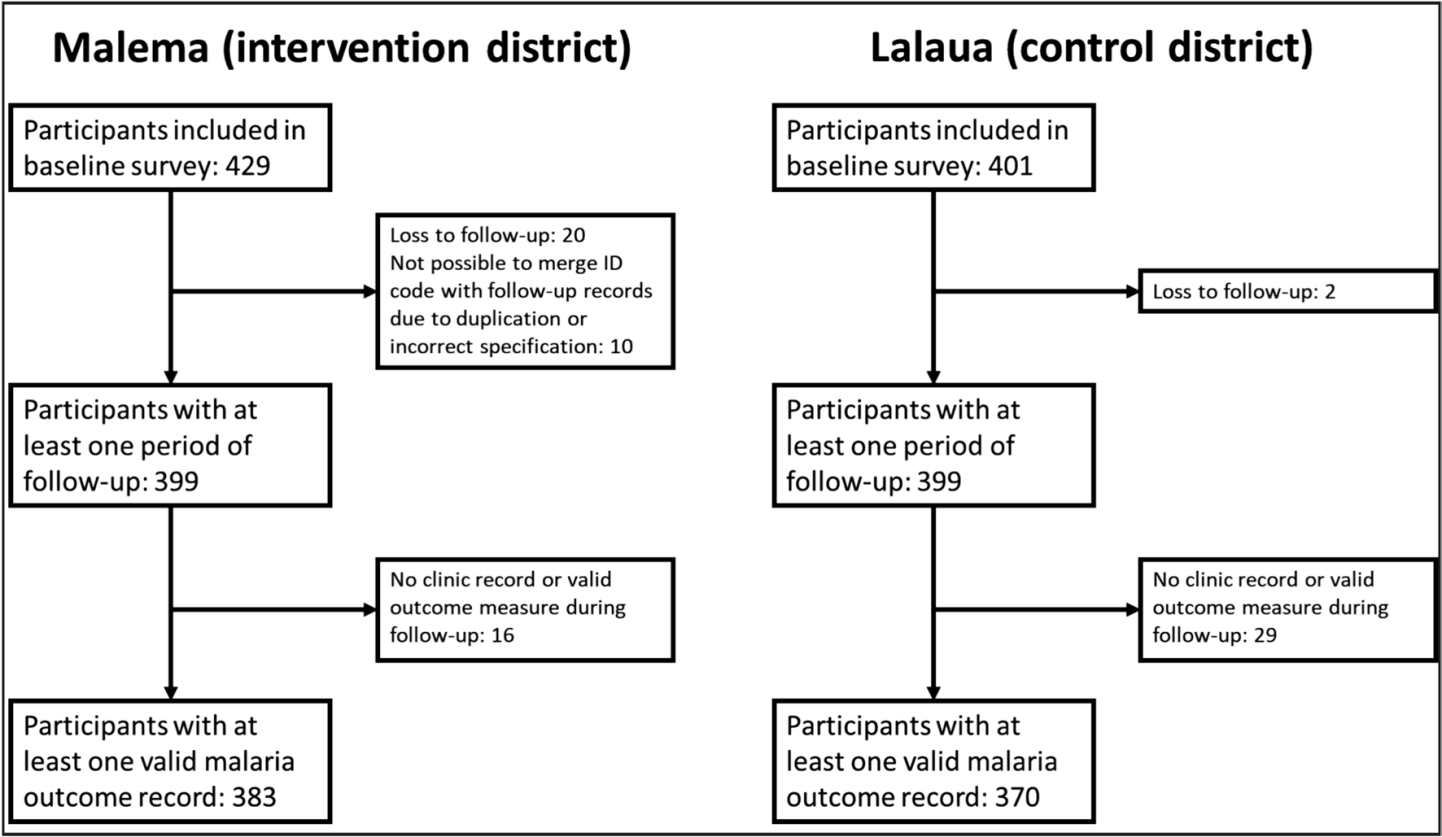
Trial profile

The baseline questionnaire was administered to a total of 830 children, as shown in Fig. 3 (429 in Malema and 401 in Lalaua). Due to loss of follow-up, no clinic record, and difficulties for matching the ID code with follow-up records, the final number of records available was 753 (383 in Malema and 370 in Lalaua).

The characteristics of the respondents, based on data collected from the baseline survey, are summarized in Table 1 for each district individually and for the two districts combined. Chi square tests are shown for the difference in distribution of responses by variable categories between the two districts. The table also shows receipt of a full course of SPAQ for each cycle. The results of the Chi square analyses showed that, at the 95% confidence level, a significantly higher proportion of participating children used a mosquito net the night before the survey and lived in a household using other malaria prevention measures before the baseline survey in the intervention district compared with the comparison district (80.7% vs. 55.7%). A significantly lower proportion of children experienced fever in the 30 days before the baseline survey based on caregiver reports (31.6% vs. 41.6%), although there was no difference in the proportions that received an anti-malarial in the same period.

**Table 1:** Participant characteristics

Variable/category	Malema (intervention)		Lalaua (comparison)		Total		Chi square test for difference between districts		
	n	%	n	%	N	%	χ2	df	p
District									
Malema	N/A		N/A		383	50.9	N/A		
Lalaua					370	49.1			
Sex									
Male	186	48.6	157	42.4	343	45.6	2.85	1	0.091
Female	197	51.4	213	57.6	410	54.4			
Child's age at baseline survey									
3–5 months	47	12.3	43	11.6	90	12.0	7.71	5	0.173
6–11 months	2	0.5	3	0.8	5	0.7			
12–23 months	80	20.9	97	26.2	177	23.5			
24–35 months	113	29.5	80	21.6	193	25.6			
36–47 months	87	22.7	94	25.4	181	24.0			
48–59 months	54	14.1	53	14.3	107	14.2			
Household mosquito net ownership									
None	76	19.8	58	15.7	134	17.8	2.78	2	0.249
One	105	27.4	98	26.5	203	27.0			
Two or more	202	52.7	214	57.8	416	55.2			
Child slept under net night before baseline survey									
Yes	309	80.7	206	55.7	515	68.4	54.43	1	< 0.001
No	74	19.3	164	44.3	218	31.6			
Net used night before survey LLIN or impregnated with insecticide in last 12 months									
Yes	267	69.7	192	51.9	459	89.1	0.515	1	0.473
No	12	3.1	6	1.6	18	3.5			
Respondent does not know	26	6.8	1	0.3	27	5.2			
Missing	78	20.4	171	46.2	11	2.1			
Household structure received indoor residual spray within last 12 months									
Yes	11	2.9	3	0.8	14	89.1	4.96	1	0.026
No	345	90.1	366	98.9	711	3.5			
Respondent does not know	26	6.8	0	0.0	26	5.2			
Missing	1	0.3	1	0.3	2	2.1			
Use of other preventive measures against malaria* night before baseline survey									
Yes	28	7.3	8	2.2	36	4.8	10.96	1	0.001
No	355	92.7	362	97.8	717	95.2			
Wealth index (number of household assets) *									
0–1	91	23.8	121	32.7	212	28.2	24.22	3	< 0.001
2–3	115	30.0	152	41.1	267	35.5			
4–9	146	38.1	87	23.5	233	30.9			
Missing	31	8.1	10	2.7	41	5.4			
Child had fever in previous 30 days before baseline survey									
Yes	121	31.6	154	41.6	275	36.5	8.41	1	0.004
No	258	67.4	211	57.0	469	62.3			
Respondent does not know	2	0.5	4	1.1	6	0.8			
Missing	2	0.5	1	0.3	3	0.4			
Child received anti-malarials in previous 30 days before baseline survey									
Yes	101	26.4	100	27.0	201	26.7	1.22	1	0.727
No	275	71.8	261	70.5	536	71.2			
Respondent does not know	2	0.5	0	0.0	2	0.3			
Missing	5	1.3	9	2.4	14	1.9			
Child received Day 2 and Day 3 AQ doses, cycle 1									
Yes	329	85.9	N/A		N/A		N/A		
No	2	0.5							
Missing	52	13.6							
Child received Day 2 and Day 3 AQ doses, cycle 2									
Yes	341	89.0	N/A		N/A		N/A		
No	1	0.3							
Missing	41	10.7							
Child received Day 2 and Day 3 AQ doses, cycle 3									
Yes	349	91.1	N/A		N/A		N/A		
No	5	1.3							
Missing	29	7.6							
Child received Day 2 and Day 3 AQ doses, cycle 4									
Yes	300	78.3	N/A		N/A		N/A		
No	9	2.3							
Missing	74	19.3							

^*^For the purposes of analysis wealth index was fitted as a continuous variable. Median index value among participating children was 3 (interquartile range: 1–4). Calculation of Cronbach’s alpha for the index gave a value of 0.59, indicating borderline-acceptable internal consistency

468 (62.2%) of 753 participants had full follow-up over the 4-month study period between the time of the baseline survey and the end of the month following delivery of the final SMC cycle. Meanwhile, 285 (37.8%) had at least 1 month of follow-up missing, of which 210 1 month and 75 2 or 3 months.

Including recurrent cases, participants in the intervention arm experienced a total of 62 confirmed cases over 1335 person-months of follow-up (implied incidence: 0.05 cases per child-month). In the comparison district there were 278 cases over a total of 1288 person-months of follow-up (implied incidence: 0.22 cases per child-month). There were no reported instances of mortality among participating children during the follow-up period.

Data on malaria cases among participating children during follow-up found that, of the 383 participants in the intervention arm, 57 (14.9%) experienced at least one RDT-confirmed case of malaria during the follow-up period while 326 (85.1%) did not; meanwhile, in the comparison arm, 210 (56.8%) of 370 participants experienced an RDT-confirmed malaria case while 160(43.2%) did not. The results of the Pearson Chi square test found a significant difference in the proportion of children experiencing an RDT-confirmed malaria case (χ^2^ = 144.19, df = 1, p < 0.001). Participants in the intervention arm had 87% lower odds of experiencing an RDT-confirmed malaria case than in the comparison arm (without consideration of recurrent cases or individual duration of follow-up) (OR 0.13, 95% CI 0.09–0.19, p < 0.001).

**Table 2:** Resistance to sulfadoxine-pyrimethamine and amodiaquine: Plasmodium falciparum pfdhps, pfdhfr, Pfmdr1 and Pfcrt polymorphism frequencies by arm for each period

Gene	SNPs*	Intervention			Control		
		Baseline n (%)	Endline n (%)	*p*^†^	Baseline n (%)	Endline n (%)	*p*^†^
*Pfdhfr*	N51I			0.340			0.914
	mutant	150 (100)	167 (100)		171 (100)	173(100)	
	total (n)	150	167		171	173	
	C59R			0.340			0.914
	mutant	150 (100)	167 (100)		171 (100)	173(100)	
	total (n)	150	167		171	173	
	S108N			0.340			0.914
	mutant	150 (100)	167 (100)		171 (100)	173(100)	
	total (n)	150	167		171	173	
	I164L			0.340			0.914
	mutant	150 (100)	167 (100)		171 (100)	173(100)	
	total (n)	150	167		171	173	
*Pfdhps*	I431V			0.618			0.558
	mutant	3 (1.2)	2 (0.8)		2 (0.7)	3 (1.2)	
	total (n)	243	254		289	256	
	A437G			0.111			0.971
	mutant	207 (85.2)	231 (89.9)		258 (89.3)	227 (89.4)	
	total (n)	243	257		289	254	
	K540E			0.292			0.559
	mutant	153 (63.7)	172 (68.3)		188 (65.1)	161 (62.6)	
	total (n)	240	252		289	257	
	581G			0.614			0.057
	mutant	3 (1.2)	2 (0.8)		4 (1.4)	0 (0.0)	
	total (n)	241	253		285	255	
	A613 S/T			0.378			0.518
	mutant	4 (1.6)	2 (0.8)		1 (0.4)	2 (0.8)	
	total (n)	243	255		282	261	
*Pfmdr1*	N86Y			0.426			0.150
	mutant	0 (0.0)	0 (0.0)		0 (0.0)	0 (0.0)	
	total (n)	247	265		296	262	
	Y184F			0.418			0.770
	mutant	134 (55.1)	134 (51.5)		160 (54.8)	136 (53.5)	
	total (n)	243	260		292	254	
*Pfcrt*	K76T			0.365			0.494
	mutant	0 (0.0)	0 (0.0)		0 (0.0)	0 (0.0)	
	total (n)	188	206		216	202	
	C72S						
	mutant	0 (0.0)	0 (0.0)		0 (0.0)	0 (0.0)	
	total (n)	188	206		216	202	
	V73						
	mutant	0 (0.0)	0 (0.0)		0 (0.0)	0 (0.0)	
	total (n)	188	206		216	202	
	M74I						
	mutant	0 (0.0)	0 (0.0)		0 (0.0)	0 (0.0)	
	total (n)	188	206		216	202	
	N75E						
	mutant	0 (0.0)	0 (0.0)		0 (0.0)	0 (0.0)	
	total (n)	188	206		216	202	
*Pfmdr1_R1*	N86Y			0.426			0.150
	mutant	0 (0.0)	0 (0.0)		0 (0.0)	0 (0.0)	
	total (n)	247	265		296	262	
	Y184F			0.418			0.770
	mutant	134 (54.25)	134 (50.56)		160 (54.8)	136 (53.5)	
	total (n)	247	265		292	254	
*Pfmdr_R2*	D1246Y			0.975			
	mutant	1 (0.44)	0 (0.0)		0 (0.0)	0 (0.0)	
	total (n)	225	239		262	237	

^†^Chi-squared test

^*^SNP single-nucleotide polymorphism

Results of Cox proportional hazards models showed that the hazard of RDT-confirmed malaria cases was significantly lower in the intervention district than the comparison district. The analysis found that none of the covariates considered significantly improved model fit for the three models, and unadjusted models were therefore fitted in all instances. Inspection of Schoenfeld residuals for Model 1 indicated that the proportional hazards assumption was met. Models 2 and 3 for recurrent events included data from 753 respondents over 1,052 periods of follow-up of a total of 84,325 child-days.

**Fig. 4:**
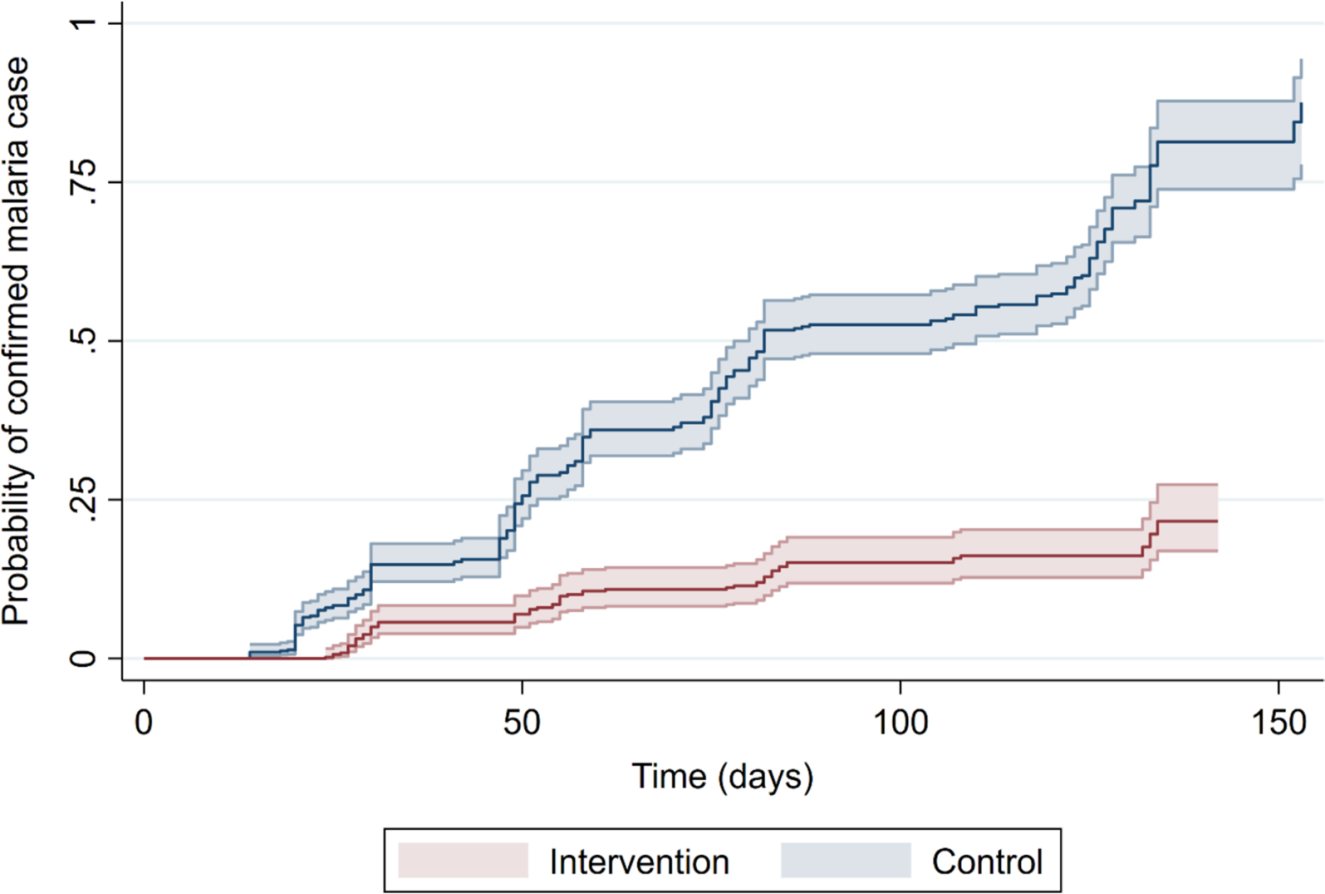
Kaplan–Meier graph intervention (Malema) and comparison (Lalaua) districts (Model 2)

Model 1 for RDT-confirmed malaria cases during the first period of follow-up gave a HR of 0.18 (95% CI 0.14–0.23), indicating that the hazard of RDT-confirmed malaria cases was 82% lower in the intervention district compared with the comparison district. The results of Model 2 for recurrent events gave a HR of 0.16 (95% CI 0.11–0.21), indicating an effect size of 84%. Finally, the results of Model 3 for recurrent events with random intercepts fitted for settlements gave a HR of 0.14 (95% CI 0.09–0.24). The Kaplan–Meier graph (Fig. 4) based on results of Model 2 shows the probability of RDT-confirmed malaria cases in the intervention and comparison districts by time of follow-up.

### Adverse events

There were no serious adverse events identified by the research team during or after the administration of SPAQ in each of the four cycles. However, 18/429 (4.2%) adverse events were recorded, with vomiting and abdominal pain the most frequently reported.

### Drug resistance molecular markers study

A total of 1198 dry blood spots were collected and genotyped: 598 samples during the baseline survey, and 600 during the endline survey. The baseline prevalence of *Pfdhps* A437G and K540E was above 60% in both control and intervention areas. When comparing the baseline and endline marker frequencies, a non-statistically significant trend of increasing prevalence of A437G and K540E *Pfdhps* single-nucleotide polymorphism (SNPs) was observed in the intervention arm, with increases from 85.2–89.9% (*p* = *0.1*) to 63.7–68.3% *(p* = *0.3)*, respectively (Table 2). The prevalence of *pfdhfr* gene mutations of N51I, C59R and S108N in both arms at baseline and endline was 100%. No mutations were found for I164L (Table 2).

**Table 3:** Mutation combination frequencies by arm and timepoint

Mutation	SNPs*	Intervention			Control		
		Baseline n (%)	Endline n (%)	*p*^†^	Baseline n (%)	Endline n (%)	*p*^†^
*Pf* dhps double	A437G & K540E (n)	236	250	0.221	280	252	0.559
	Yes	148 (62.7)	170 (68.0)		178 (63.6)	154 (61.1)	
	No	88 (37.3)	80 (32.0)		102 (36.4)	98 (38.9)	
*Pf* dhps triple	A437G, K540E & A581G (n)	234	246	0.960	275	250	0.340
	Yes	2 (0.9)	2 (0.1)		1 (0.4)	0 (0.0)	
	No	232 (99.1)	244 (99.2)		274 (99.6)	250 (100)	
Pf dhfr double	N51IR & S108N (n)	150	167	0.340	171	173	0.914
	Yes	150 (100)	167 (100)		171 (100)	173 (100)	
	No	0 (0.0)	0 (0.0)		0 (0.0)	0 (0.0)	
Quintuple	A437G, K540E, N51I, C59R & S108N (n)	142	144	0.736	159	153	0.096
	Yes	86 (60.6)	90 (62.5)		106 (66.7)	88 (57.5)	
	No	56 (39.4)	54 (37.5)		53 (33.3)	65 (42.5)	
Sextuple	A437G, K540E, A581G, N51I, C59R & S108N (n)	141	143	0.992	156	153	0.865
	Yes	1 (0.7)	1 (0.7)		0 (0.0)	0 (0.0)	
	No	140 (99.3)	142 (99.3)		156 (100)	153 (100)	

^†^Chi-squared test

^*^SNP single-nucleotide polymorphism

There were no mutations for mdr1_R1 N86Y, and prevalence of mutations for mdr1_R1 Y184F was similar across arms and between baseline and endline (55.1%, 51.5%; p = 0.418) for the intervention arm vs (54.8, 53.5; p = 0.770) for the control arm. There was no evidence to suggest that the frequency of any of the resistance markers analysed during the study was significantly higher after the intervention, since all *p*-values were above 0.1.

*Pfdhps* triple mutation (A437G/K540E/A581G) was detected in 0.9% of the samples from the intervention arm at the baseline, compared to 0.4% of those at baseline in the comparison arm. Sextuple combination was observed only in the intervention arm (0.7%). There was no difference between the baseline and endline (n = 1) (Table 3).

**Table 4:** Characteristics of IDI with key informants

Level		Sex	Number of interviewees
Central	Maputo	Female	1
		Male	3
		Subtotal	4
Province	Nampula	Male	3
		Female	1
		Subtotal	4
District	Malema	Male	5
		Female	1
	Mecubúri	Male	5
		Female	1
	Subtotal		12
TOTAL			20

### End-of-round survey

The coverage survey found that 85.8% (95% CI 82.1–88.9) of eligible children aged 3–59 months received Day 1 SPAQ in cycle 4 in Malema and Mecubúri combined; of these 96.1% (95% CI 93.7–97.6) received SPAQ from distributors adhering to DOT and 98.3% (95% CI 98.5–99.7) received AQ on Day 2 and Day 3 administered by caregivers. In addition, 77.0% (95% CI 69.7–82.9) received Day 1 SPAQ in all four cycles. Results from the representative sample of ineligible older children aged 60–119 months found that, based on caregiver self-reporting, 15.3% (915.3 (95% CI 11.5–20.1) received Day 1 SPAQ in cycle 4.

### SMC acceptability and feasibility

Main characteristics of FGDs and IDIs participants are shown in Tables 4 and 5.

**Table 5:** Characteristics of FGDs participants

Target population	District	Sex of participants	Nr of participants	Age (range)	Area		Number of FGDs
					Peri-urban	Rural	
Caregivers	Malema	Female	6	18–24 years	1	1	2
		Female	7	> 25 years	1	1	2
		Male	7	> 18 years	1	1	2
	Mecubúri	Female	5	18–24 years	1	1	2
		Female	6	> 25 years	1	1	2
		Male	6	> 18 years	1	1	2
	Total						12
CD^a^	Malema	Female	6	> 25 years	1	1	2
	Mecubúri	Male	7	> 18 years	1	1	2
	Total						4
CD’s supervisor	Malema	Male/Female	5	> 18 years	1	1	2
	Mecubúri	Male/Female	6	> 18 years	1	1	2
	Total						4

^a^*CD* Community Distributor

### Knowledge about SMC

Caregivers described finding out about the SMC campaign in a variety of ways. Some caregivers reported hearing about SMC from the Community Health Workers (CHWs), who provided information to the communities they were covering and addressed their questions. Other caregivers reported becoming aware of SMC from community leaders in their church, who received training prior to the intervention on how to spread and provide information about SMC. Some caregivers also heard of SMC trough radio and television messaging. As information was being spread in the intervention areas, some caregivers also reported learning about SMC from their neighbourhoods during their work in the fields.

### Benefits of SMC

In general, participants had positive views and opinions concerning SMC, which was perceived as an effective intervention at preventing malaria, and consequently improving the health of their children. Caregivers also recognized that good malaria prevention directly affected their quality of life, reducing their time in health facilities for treatment. Caregivers, and in particular mothers, also reported SMC improving the quality of their and their families’ lives by allowing them to invest their energy in other household chores or agricultural practices, and by reducing demand for healthcare. The interviewed stakeholders perceived the SMC campaign as helpful in controlling and reducing cases of malaria in the population, especially when compared with the same period of the year in the past.

*“…this is what I was saying, that is before they brought these pills [here in the community] we left our activities because the children got sick all the time and then they [their mothers] took them to the hospital that it already created a absence (of work) in our fields. Now that they* [the community distributors] *gave the pills and our children already play, and we already have strength, that is when we saw that life is like this.”*(Caregiver, female, rural area)

*“I think that if this strategy remains as a routine activity, an activity for the whole country, it will help a lot to prevent malaria widely, reducing it largely for that group included in this activity, we had children under our monitoring.”* (Stakeholder, male, rural area)

Community distributor supervisors at facility level also perceived that the intervention was having a positive impact among the population that received the intervention. They noticed a reduction in the number of malaria cases, while the incidence of other diseases remained high. Community distributor supervisors also reported noticing a change in behaviour across communities, moving from initial hesitancy against the intervention to embracing its positive effects.

### Challenges in implementation

Participants reported some challenges in accepting SMC during the initial roll out of the campaign. Among the most frequently reported barriers they mentioned general mistrust, lack of partner approval, possible side effects of the medicines, and local beliefs about the treatment. Vomiting was the most frequently reported adverse reaction.

*“We are used to going to the hospital, this thing of bringing pills to our homes without getting sick started to happen. I never saw pills distributed in homes, is the first time. They [those who did not participate to the campaign] were afraid of how it is possible to distribute pills in homes to children who are not even sick, so this generated fear.”* (Caregiver, female, rural area)

*“…cultural issues, first, there are certain religions in which the child or any family member is not allowed to take the medication or any medication. They rejected it, was not easy”.*(Community distributor supervisor, peri urban area)

Community distributors’ supervisors also mentioned challenges related to Covid-19, linking the misinformation concerning the pandemic with a general mistrust of medicines among the community. At times, cultural and religious beliefs motivated a refusal of the medication.

*"... it was a challenge at a time when, in the middle of a pandemic, it ended up creating a feeling of fear when we look at the community uneducated people who at some points are always distrust.”* (Community distributor supervisor, peri-urban area*)*

*The second issue was because it was a new strategy, not even at the national level, it was only implemented in two Districts of Nampula province. Thus, some people didn't want them to be, they thought they were people who were being used for an experiment so at some point they were limited they suffered from agitation a lot of misinformation.”* (Community distributor supervisor, peri urban area*)*

Stakeholders at provincial level communicated that the payment model and delay of payments to implementers posed challenges during the SMC campaign. As many implementers do not have mobile phones and network in rural areas is often weak, processing mobile payments was challenging.

*"…The method adopted for payment, especially for implementers, does not reflect the reality of our communities, many distributors they do not have the SIM card that was a condition for payment by mobile model.”* (Key Informant, Provincial level)

### Facilitators of adherence to the SMC protocol

Caregivers valued the involvement of members of their own community in providing information, entering their homes, and distributing the medicines to their children during the SMC campaign. In many cases it was reported that the inclusion of community leaders, religious leaders and people speaking Emakhuwa helped caregivers who in the beginning declared themselves against the SMC campaign to decide to accept the intervention. Observing the high acceptance by other members of the community, lack of adverse effects on children and the positive feedback given by their neighbourhoods also helped to convince hesitant caregivers.

*“What made me accept the campaign and participating in it was the fact that the individuals involved were not foreigners [of the community], at first, I thought the intention was to harm the health of our children and leave... But as the people involved in the campaign are people we know, and we know where to look for them and find them in case of any health problem in children. All in all, the campaign went smoothly, and we feel that our children are in good health and malaria cases have greatly reduced in our community.”* (Caregivers, female, rural area)

*“Those who were afraid [of SMC] asked in the neighbourhoods the reaction of the children after taking them [the pills] and we were informed that we did not see anything serious and that they could also receive [the pills] from that moment the persons started participating too.”* (Caregivers, female, rural area)

*“And we also gained the courage to accept our children being given the medicines because they* [the community distributors] *were always accompanied by the secretary of our community.* (Caregiver, female, semi urban area)

Community distributors and their supervisors expressed the importance of engaging local and religious leaders in community mobilization during the SMC campaign. SMC implementers worked together with community leaders who acted as guides in the communities. Key informants mentioned that the good coordination between local and national level institutions contributed to the successful implementation of SMC. The involvement of community members in the SMC campaign as community distributors and in other key positions positively impacted the acceptance of the intervention among the communities.

*“The personnel involved as community distributors was recruited at the local level, they are sons of those communities, and the mobilizers were community leaders from their respective communities.”* (Stakeholder, Health District Services)

### Future implementation of SMC

Participants provided various suggestions about the future implementation of SMC campaigns. Those suggestions included expanding the age of the intervention’s target population to older children and adults. They suggested that larger scale deployment of SMC should be the next step.

*“In our opinion, that in the next years, the campaign for the distribution and administration of medicines should be more comprehensive, because adults are also vulnerable to diseases such as malaria.”* (CG, female, rural area)

*“… knowing that the disease, when it comes, covers all children, these are the difficulties we have, so we are asking to bring it for children from 6 to 10 we are asking too.”* (CG, female, semi urban area)

## Discussion

Study findings suggest that SMC is effective in preventing clinical malaria among children 3–59 months during the high transmission season in northern Mozambique. Results of the nRCT show an estimated protective effect of SMC of 86%. This reflects similar results shown in a recent study of similar design conducted in Uganda [18], which found a protective effect of 92%, and with the pooled result of a case–control study conducted in five countries showing a protective effect of 88% [19]. The observed results also align with those of other clinical trials showing the high coverage achieved by SMC, and reporting good adherence at day 2 and 3, as per results of the EoR coverage survey conducted in this study [16]. Large scale clinical trials administering SP + AQ as SMC [20–22] have shown significant protective effectiveness against clinical malaria during the transmission season. While some work has been done [23], the impact of seasonality on SMC effectiveness needs to be fully understood in east and southern Africa, and further work is required to document this, especially as new locations in ESA, including these study sites, do not necessarily completely fit the WHO seasonality criteria for SMC. Some recent work done using dynamical modelling found that the effect size of SMC is highest when baseline incidence is lowest, suggesting the need to account for seasonality in programming [24].

During the implementation of SMC in four cycles the most frequent adverse event reported was vomiting and fever. These adverse effects were also found as the most frequently reported in other studies [25–27]. All the adverse events were resolved without any other intervention or administration of other medications.

The absence of *Pfdhfr* 164L marker is reassuring as this is often associated with pyrimethamine failure [28]. Similarly, no *Pfrcrt* mutations were detected, suggesting absence of amodiaquine resistance mediated by *Pfcrt*. *Pfmdr1* mutants are found in approximately half of the samples processed for 184F. Change over one round of any marker is not significant and based on the data presented here, there is no statistical evidence that the observed difference is due to SMC introduction. The combination of SNPs of relevant *Pfdhps-dhfr* mutants is notable. Also, finding no 86Y or 76 T mutations is notable and could mean that there has been full reversion of CQ sensitivity, and thus AQ sensitivity, in this area. Further analysis is being done to understand what extent the effect of SMC is driven by AQ, versus the contribution of SP, and the results of this analysis will be presented in future work. Long term implications of this observation will be much better understood once the molecular and chemoprevention efficacy component results of phase 2 study become available.

The study also revealed a generally high acceptance of SMC among communities, with caregivers reporting a significant reduction in malaria among their children and an improvement in their quality of life. Community members learned about SMC campaign from different sources of information such as community health workers, their leaders, their neighbours, and mass media. This multi-source approach in spreading the information about SMC and its objectives in this context worked effectively. The involvement of community members has been identified as a key advantage in delivering SMC and malaria messages at community level by other studies [29–31]. Benefits of SMC have been already reported by caregivers of children less than five years of age, holding the view that the combination drug was very useful in preventing malaria [32, 33]. Despite the general acceptance and positive perception of SMC, caregivers, community distributors and stakeholders reported several challenges, such as mistrust, lack of partner approval, fear for side effects and local beliefs. These barriers have been also reported in other contexts when delivering a community intervention [33]. However, it has been suggested that continued health education can increase the acceptability of SMC [32], as well as to drive the delivery method and applying the earned trust [30] and the delay in payment incentives to staff [33]. On the other hand, caregivers and key informants provided clear views about enablers for the acceptability of the campaign, that as already noted here above were the involvement of community members and community leaders, both in spreading the information and distributing the medication, the door-to- door delivery, the free access to the medication and the community network, as observed also in other contexts [30, 34]. Participants’ suggestion to expand the intervention to older age groups illustrates the high acceptability of the intervention and reflects the perception on the burden of malaria as a health concern that affects not only young children but also older age groups. Some studies indicate that expanding SMC to older children can contribute towards reducing the incidence of malaria [20, 25], and the most recent WHO guidelines for malaria recognize that the target age for SMC should be selected based on risk of severe malaria [1]. Overall, these results provide valuable insights into the implementation of SMC at community level, emphasizing the importance of involving the community, utilizing the natural network to increase the transparency about the objectives and the ownership of all the beneficiaries.

### Strengths

The study design includes a variety of methods, combining a non-randomized controlled trial, cross-sectional surveys, and qualitative interviews. This broad scope allows for a comprehensive, initial understanding of the intervention’s effects. Secondly, the inclusion of a comparison (Lalaua) and intervention districts (Malema and Mecubúri) allows for comparison, increasing the reliability of the results. Thirdly, the follow-up visits after each cycle and detailed recording of confirmed malaria cases provide robustness to the study’s ability to accurately assess the intervention. Finally, the inclusion of a diverse range of stakeholders at any level up, from the community to the central level, provides a more exhaustive perspective of the feasibility and acceptability of SMC.

### Limitations

Despite the encouraging results provided by this study, however there are several limitations. First, the absence of randomization in the trial design may have introduced bias as the arms were not evenly balanced with respect to potential confounding factors. However, after analysis of baseline characteristics of children, caregivers, and households in the two follow-up groups, differences were found in only two variables: children’s use of bednets the night before the base line survey and incidence of fever in the previous month before the baseline survey among children. This baseline imbalance was addressed by use of random effects at the community level to account for community-level differences in malaria transmission. Also, as there was some loss to follow-up (~ 10%), which could have impacted on the overall power of the final analysis; however, the study was powered conservatively to detect a significant difference in hazard of malaria cases of only 40%. Secondly, bias may have been introduced in the qualitative data through the selection of respondents who had received SMC. Thirdly, caregivers’ report on fever or adherence on day 2 and 3 may have introduced recall bias in the way caregivers reported the following month to community distributors, especially if caregiver’s recall is influenced by their perceptions of SMC. Fourthly, this study was conducted in a specific area of Nampula province, affecting the generalizability of the findings to other regions of Mozambique or other countries. This will be addressed by further studies, such as the rapid assessments [35]. Finally, Sanger sequencing was used to analyse molecular markers of anti-malarial resistance, which has lower sensitivity to detect mixed infections (resistant and mutant alleles) when compared to next generation sequencing.

## Conclusion

This study’s encouraging results marks the first attempt to test the suitability of SMC as a malaria prevention strategy in east and southern Africa, where malaria transmission is seasonal but resistance to SPAQ is high. Results from this study and a similar study in Uganda support the potential deployment of SMC in new geographies outside of the Sahel. Future research on the use of SMC in east and southern Africa will explore alternative drug regimens as well as the cost-effectiveness of SMC when implemented at scale in the context of high resistance. Phase 2 of this study will determine the effectiveness of SMC using a randomized design, as well as the chemoprevention efficacy of SPAQ in new areas such as these to determine timelines as to how long SMC using SPAQ is likely to retain its current effectiveness despite drug resistance. In addition, similar assessments will be required in traditional geographies to accurately predict future effectiveness. Further investigation is also needed into the role of climate change and changing seasonality patterns, how SMC impacts immunity, the implications of introducing SMC on first-line malaria treatment and other malaria prevention interventions, particularly other forms of chemoprevention as well as interactions with other diseases or health issues.

## Data Availability

The associated study protocol and data collection tools will be made available upon request from the corresponding author. Quantitative datasets are available from the corresponding author upon reasonable request after the completion of primary analyses and results dissemination. Qualitative study datasets will not be available, as they may include identifiable information that could compromise participant confidentiality.

## Abbreviations

AQ: Amodiaquine
CHW: Community Health Worker
DHFR: Dihydrofolate reductasee
DHPS: Dihydropteroate synthetase
DOT: Directly observed therapy
EoR: End-of-Round
FGD: Focus group discussion
KII: Key informant interview
NMCP: National Malaria Control Programme
*Pfcrt*: Plasmodium falciparum chloroquine resistance transporter gene
*Pfmdr1*: Plasmodium falciparum multidrug resistance gene 1
nRCT: Non-randomized controlled trial
SMC: Seasonal malaria chemoprevention
SP: Sulfadoxine-pyrimethamine
SPAQ: Sulfadoxine-pyrimethamine and amodiaquine
WHO: World Health Organization
RDT: Rapid diagnostic test
